# Growth Mindset Moderates the Effect of the Neonatal Resuscitation Program on Performance in a Computer-Based Game Training Simulation

**DOI:** 10.3389/fped.2018.00195

**Published:** 2018-07-04

**Authors:** Maria Cutumisu, Matthew R. G. Brown, Caroline Fray, Georg M. Schmölzer

**Affiliations:** ^1^Centre for Research in Applied Measurement and Evaluation, University of Alberta, Edmonton, AB, Canada; ^2^Neonatal Research Unit, Centre for the Studies of Asphyxia and Resuscitation, Royal Alexandra Hospital, Alberta Health Services, Edmonton, AB, Canada; ^3^Department of Educational Psychology, Faculty of Education, University of Alberta, Edmonton, AB, Canada; ^4^Department of Computing Science, University of Alberta, Edmonton, AB, Canada; ^5^Division of Neonatology, Department of Pediatrics, University of Alberta, Edmonton, AB, Canada

**Keywords:** newborn, delivery room, neonatal resuscitation, growth mindset, performance, neonatal resuscitation program, serious games, computer-based game simulation

## Abstract

This study examines for the first time the moderating role of growth mindset on the association between the time elapsed since participants' last refresher neonatal resuscitation program (NRP) course and their performance on neonatal resuscitation tasks in the RETAIN computer game training simulation. Participants were *n* = 50 health-care providers affiliated with a large university hospital. Results revealed that growth mindset moderated the relation between participants' task performance in the game and the time since their latest refresher NRP course. Specifically, participants who completed the course more recently (i.e., between 8 and 9 months before the current study) made significantly more mistakes in the game than the rest of the participants but only when they endorsed lower levels of growth mindset. Implications of this research include growth mindset interventions and increased screen time in simulation sessions that have the potential to help health-care providers achieve better performance on neonatal resuscitation clinical tasks.

## Introduction

The delivery room is a stressful environment where decisions are made quickly and resuscitators must have good cognitive, psychomotor, and communication skills to recognize, analyze, and integrate a large amount of data into useful information under intense time pressure. Several studies reported that the complexity of these tasks leads to deviations from the Neonatal Resuscitation Program (NRP) algorithm, human errors, and poor patient outcomes during simulated neonatal resuscitation (1–7). Studies also reported that health-care providers (HCP) commit errors 16–55% of the time in simulated neonatal resuscitation (4, 7). McCarthy et al. analyzed 189 delivery room resuscitations and reported non-compliance with resuscitation guidelines in up to 90% of the cases (1). However, when resuscitation teams were more vigilant, they committed significantly fewer errors (8). Similarly, the Joint Commission on Accreditation of Healthcare Organizations (2004) reporting on preventing infant death and injury during delivery identified human errors during neonatal resuscitation as responsible for more than two thirds of perinatal mortality and morbidity (9).

One of the main causes of human error in neonatal resuscitation stems from a lack of practical learning experiences (10, 11) highlighted by the neonatal training paradox (12) of high-acuity, low-occurrence (HALO) situations that arise infrequently. Therefore, the recent neonatal resuscitation guidelines recommend simulation-based medical education (SBME) to enhance knowledge retention and decrease human errors in real-life clinical situations (13). Simulation-based training has been shown to be effective in preparing HCP for HALO events (10). However, it is resource and cost intensive, and the optimal frequency for development of competency and for supporting knowledge retention is unknown. Moreover, while SBME has been shown to improve performance initially after training (14, 15), both cognitive and technical skills significantly deteriorate within months (16). Therefore, other methods of training to improve knowledge retention and decision-making are needed.

Educational games foster higher-order thinking, such as analysis, synthesis and evaluation skills necessary during HALO events by enabling active-learning experiences (17, 18). Further, computer-based simulators can improve overall knowledge and decision-making, thereby reducing mistakes (17–21). Therefore, simulators are increasingly used as supplemental teaching tools in medical education. Traditional lectures often do not reflect the levels of complexity of practical applications or real-life situations. By contrast, simulators are simplified models of complex real-life systems that clarify difficult issues by presenting them as plain game processes (22). In addition, they have the potential to motivate learners. Structured and rule-guided, they provide enjoyable physical or mental training, including narrative and simulative aspects. While other areas of expertise (e.g., military education) have used simulators for centuries, the first simulators for medical undergraduate and postgraduate education were developed only in the twentieth century (23, 24). Although many simulators have been developed to address adult resuscitation or emergency situations (17, 19, 22, 25–33), the development of simulators that specifically address neonatal resuscitation are lacking.

**Figure 1:**
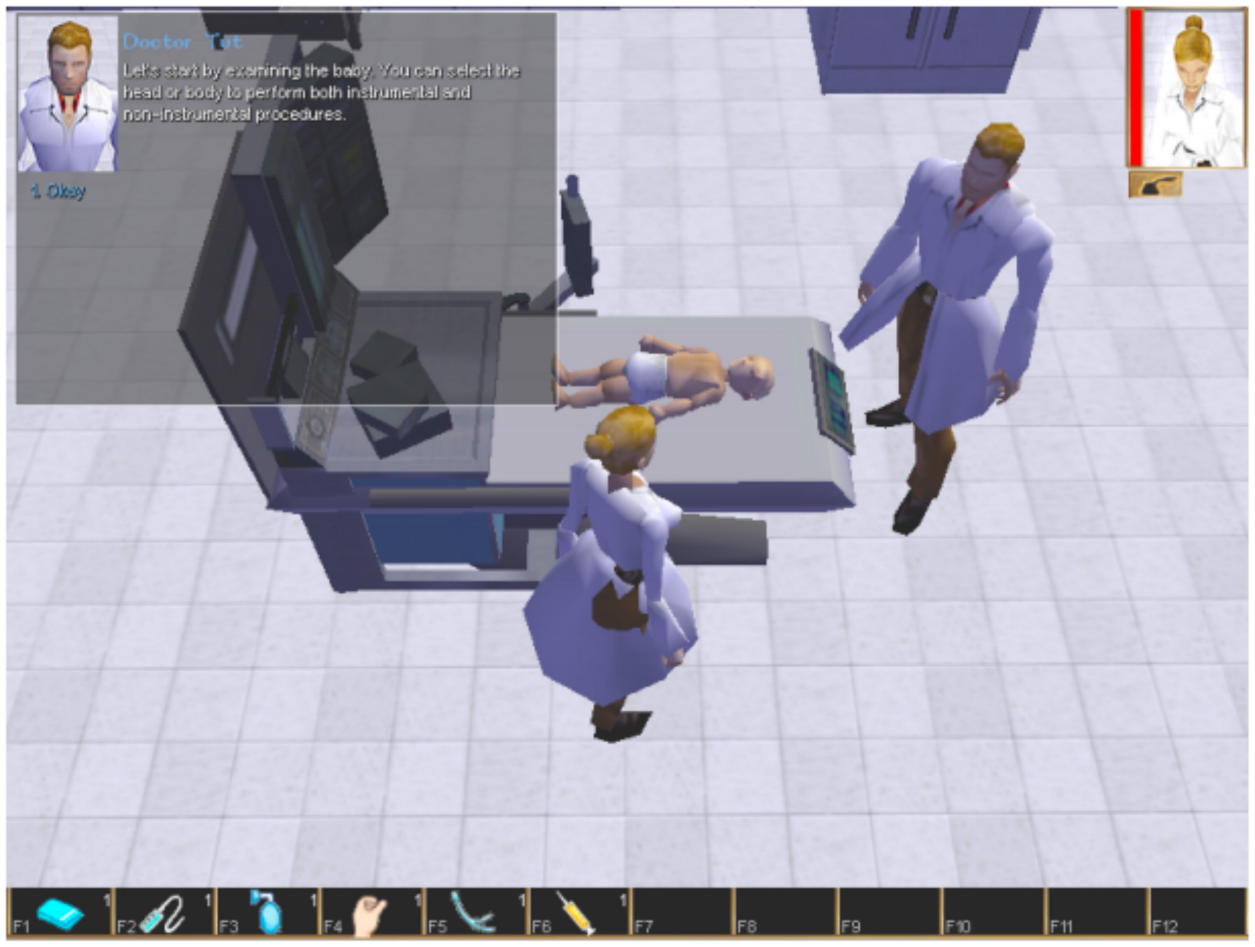
The RETAIN game: participants play through a tutorial and three rounds of the game. The game tracks players' learning analytics, including their performance and time spent on each game round.

In previous research, we developed a complementary tool to the physical SBME to improve knowledge retention during neonatal resuscitation in the delivery room. Specifically, we developed a neonatal resuscitation training computer game simulator, RETAIN, depicted in Figure 1 (34). The game was implemented using the Neverwinter Nights game engine (35). The purpose of RETAIN is to provide neonatal resuscitation training to players. The RETAIN computer-game training simulation starts with a short in-game tutorial and continues with three levels that introduce the player to various neonatal resuscitation techniques. The player can interact with game characters that can provide information about the task.

However, there is little research on the relation between simulation-based training and performance for neonatal resuscitation. Additionally, there may be many factors (e.g., mindset) that could explain the decline in performance over time after training. Growth mindset, an incremental theory of intelligence, is defined as the belief that ability can be improved with learning and effort (36–40). In contrast, fixed mindset, an entity theory of intelligence, is the belief that intelligence is a stable entity. Individuals who perceive performance as malleable, rather than due to inherent ability, may be more likely to use strategies for performance improvement (e.g., deliberate practice). However, there is no evidence in the literature that mindset influences performance in neonatal resuscitation. Hence, this study aims to elucidate this matter for the first time. We hypothesized that HCP who endorse a growth mindset would demonstrate better neonatal resuscitation performance.

## Methods

### Participants, procedure, and data sources

The study was performed at the simulation lab at the Centre for the Studies of Asphyxia and Resuscitation, Edmonton, Canada. The Centre for the Studies of Asphyxia and Resuscitation is integrated within the Neonatal Intensive Care Unit at the Royal Alexandra Hospital, Edmonton, a tertiary perinatal center admitting more than 350 infants with a birth weight of < 1,500 g to the neonatal nursery annually. HCPs trained in NRP, including registered nurses, respiratory therapists, neonatal nurse practitioners, neonatal consultants, and neonatal fellows, were recruited from the Royal Alexandra Hospital. The study was approved by the Human Research Ethics Board at the University of Alberta (Pro00064234), and consent was obtained from all HCPs prior to participation. All participants willing to participate were included in the study (i.e., no participant was excluded). Participants had no prior experience with the RETAIN computer game.

### Study setup

Each participant was asked to complete a pre-game questionnaire to obtain demographic information (e.g., last NRP-course, years of experience, etc.) and a post-game questionnaire. Each participant played the RETAIN game, which started with a tutorial followed by three levels, each presenting a resuscitation scenario (see Figure 1). Bulitko and colleagues describe the RETAIN computer game in detail (34). Within each level, there was a countdown to simulate the stress of a real-world scenario in the three levels. In Level 1, the infant had a blocked airway and only required suctioning. In Level 2, the infant required mask ventilation and chest compression but recovered after performing chest compression. Finally, in Level 3, the infant required mask ventilation, chest compression, and epinephrine but recovered afterwards. If a player made four mistakes before solving the task correctly, the player was directed automatically to the next level of the simulator and the infant died. The RETAIN simulator collected learning analytics, including behavior and performance data, while participants played the three-level game in which they solved neonatal resuscitation challenges.

After completion of the game, each participant also completed a post-game questionnaire to assess the player's mindset. For instance, two questions were probing the participant's growth mindset: How much do you agree with the following statements? (using a Likert scale from 1 = Strongly disagree to 5 = Strongly agree): *You can always change how good you are at your job* or *You can get better at your job with practice*.

### Data collection

#### Measures

*Outcome Variable: Number of Tries* measures the number of steps it took a participant to complete the game. It sums the number of tries on each of the three levels, and it ranges from 32 to 39. On each round, participants were assigned one point for each correct step. The minimum value of 32 represents a perfect performance score (i.e., no repeated tries) on all three game rounds.

*Predictor: Last NRP Course* measures the number of months since the participants' latest NRP course. It ranges from 1 to 24 months. Other potential predictors considered in this study are *Years of Experience* (ranging from 0.08 to 32 years) and *Level of Education*, which represents a participant's highest level of education (1—diploma, certificate, or other professional program; 2—Bachelor's degree; 3—After degree; 4—Master's degree; 5—MD; 6—PhD). In this sample, there were 17 participants with diploma, certificate, or other professional programs, 29 participants with a Bachelor's degree, two with after degrees, and two with MD degrees.

*Moderator: Growth Mindset* measures the belief that intelligence can be improved with learning and effort (36) and it sums the scores of each of the two growth mindset questions presented above. The possible range is 2–10 (i.e., each of the two growth mindset items ranges from one to five) but, in this study, the minimum value of this variable was seven and the maximum value was 10.

### Statistical analyses

All analyses were performed using the *R* open statistical computing environment version *v. 3.4.1* (41). This study was designed to determine the effect of the Last NRP Course predictor variable on the performance outcome (Number of Tries) across the levels of the Growth Mindset influential variable (i.e., a moderator). Multiple linear regression analyses with robust estimation were conducted to test the main effects and interaction of Last NRP Course and Growth Mindset predicting Number of Tries (42). The model employed the *rlm* function of the *MASS* package for heteroscedasticity-robust fitting of linear models using an M estimator (43) and the *f.robftest* function of the *sfsmisc* package (44) to compute a Wald test for multiple coefficients of a *rlm* object, as the variables included in this study were not normally distributed. Analyses detected and replaced outliers in the outcome variable with the sample mean. Results held with or without the removal of the outliers. All variables were continuous, thus, to probe continuous interactions more deeply, the *jtools* R package (45) was employed, which uses the *Johnson-Neyman* (J-N) technique to determine the regions of significance for simple slopes in the context of interactions in multiple linear regressions with continuous variables (i.e., when a moderator might influence the relation between a predictor and an outcome), providing a more detailed account of the moderation (i.e., interaction) effect (46–48). In contrast to the simple slopes test approach that reveals whether the predictor is associated with the outcome at a particular value of the moderator, the J-N technique finds the range of the moderator values for which the predictor has a significant association with the dependent variable. Specifically, it shows the precise regions of the continuum of Growth Mindset values for which the relation between Last NRP Course and Number of Tries (i.e., the regression slope of Number of Tries) is estimated to be statistically significantly different from zero. It also enables the generation of a meaningful plot of conditional effects of that moderator's continuum (Growth Mindset) to visualize the regions of significance that show how the relation between the predictor (Last NRP Course) and the outcome variable (Number of Tries) is constantly changing across continuous levels of the Growth Mindset variable.

First, the predictor and the moderator continuous variables were mean centered. Robust correlation analyses between the centered variables and the outcome variable were conducted using the *WRS2* package (49). Then, assumptions of the multiple linear regression analysis were tested. The *gvlma* R package (50) was used to confirm the linearity of the association between the dependent variable, the predictor, and the moderator.

**Figure 2:**
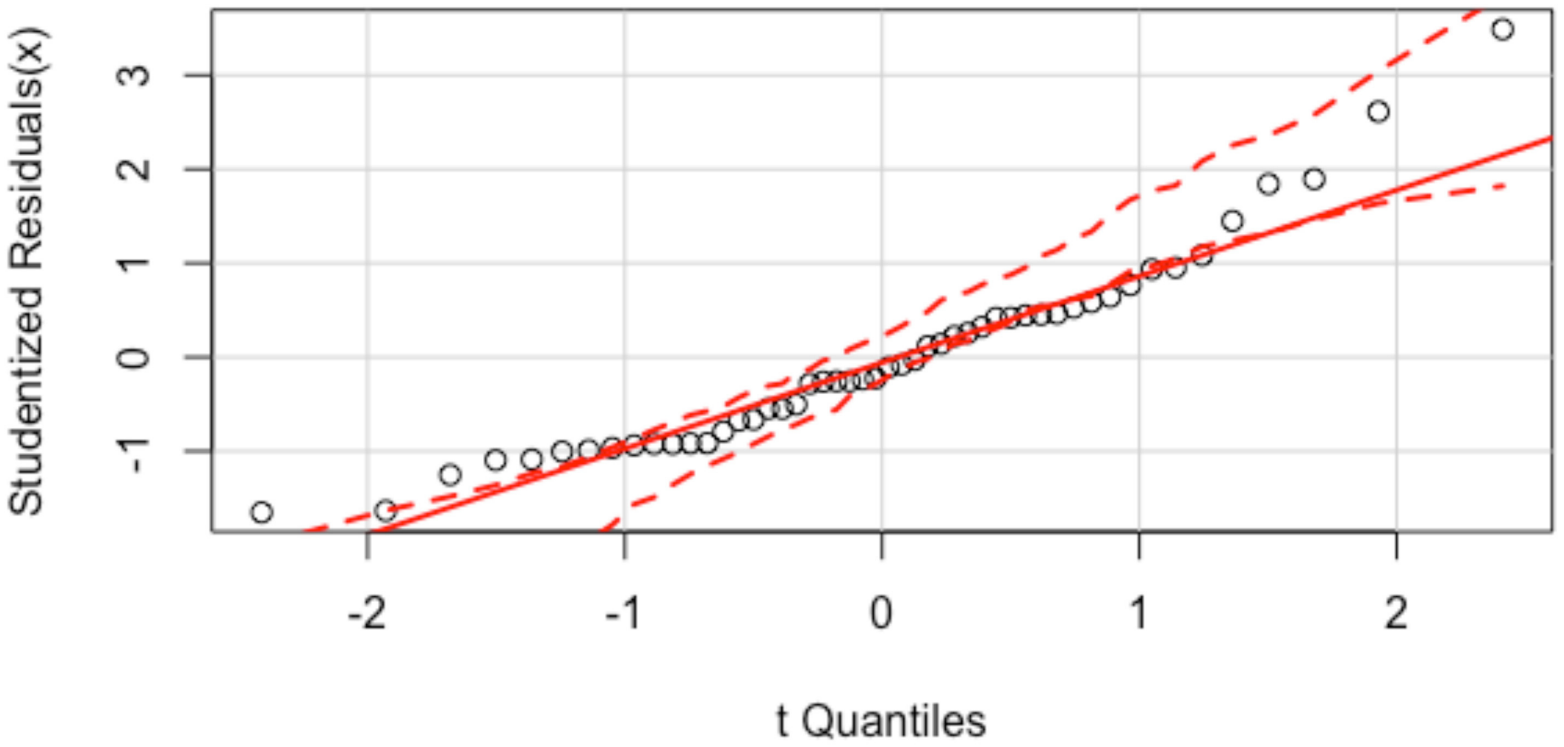
The quantile-comparison plot for the model consisting of the Last NRP Course and Growth Mindset predicting Number of Tries shows that the residuals of these variables are normally distributed, as they do not deviate from the straight solid line severely. The x-axis shows the Theoretical Quantiles and the y-axis shows the studentized residuals.

Residuals are leftover of the outcome variable after fitting a model (i.e., predictors) to data and they could reveal how well a model represents data. The Shapiro-Wilk normality test revealed that the outcome variable was not normally distributed (*W* = 0.94, *p* = 0.02). However, the residuals were normally distributed, as shown in the quantile-comparison plot of Figure 2, a graphical tool to help assess if a set of data plausibly came from a theoretical distribution (e.g., a normal or exponential distribution). Figure 2 plots empirical quantiles of studentized residuals from a linear model against theoretical quantiles of a comparison distribution. A test of non-constant variance (NCV) revealed that the variance of the residuals was constant [$\chi_{\left(1\right)}^{2}$ = 0.37, *p* = 0.54]. Multicollinearity tests yielded Variance Inflation Factor (VIF) values of 1.0, indicating that multicollinearity was not problematic.

## Results

### Descriptive analyses

We recruited 50 (45 females, 4 males, and 1 not reported) HCP who were all NRP-trained and had completed an NRP refresher course within the last 24 months of the current study. Participants needed a mean of 8.47 (*SD* = 8.66) min to complete the game. The means and standard deviations of the outcome (number of tries until completing all levels of the game), predictor (last NRP course), and moderator variables (growth mindset) are shown in Table 1. On average, participants reported high levels of growth mindset, with scores ranging from 7 to 10 and a mean of 9.17. The averages of each of the two growth mindset items that formed our Growth Mindset measure were 4.48 and 4.69, respectively. Similar mindset values were reported in a recent growth mindset intervention [i.e., an average of 4.76 out of 6; (51)]. Also, in the present study, participants took their latest NRP course 8.49 months on average prior to the current study and scored 93% on average in the RETAIN game (32 was the perfect score).

**Table 1:** Descriptive statistics of the variables included in the model before centering the predictor and the moderator.

**Measures (*n* = 50)**	**Mean (SD)**
Number of tries	34.43 (1.61)
Last NRP course	8.49 (6.01)
Level of education	1.82 (0.85)
Years of experience	9.15 (8.23)
Growth mindset	9.17 (0.93)

### Robust correlations

The robust correlations between the number of tries until completing the game, the time passed since the latest NRP course, and growth mindset are shown in Table 2. Results revealed that the more recently the participants took the NRP course, the more tries they required to complete the game.

**Table 2:** Robust correlations between growth mindset, number of tries to complete the game, and time since their latest NRP course.

**Measures (*n* = 50)**	**Last NRP course**	**Growth mindset**	**Level of education**	**Years of experience**
Number of tries	−0.29*	−0.10	0.11	0.06
Last NRP course	–	−0.001	−0.10	0.14
Growth mindset	–	–	−0.08	0.02
Level of education	–	–	–	−0.13

* *p < 0.05*.

### Multiple linear regression

The relation between time elapsed since participants' latest refresher NRP course and their game performance was explored from the lens of their theories of intelligence (e.g., growth mindset). A robust multiple linear regression was conducted to explore whether participants' growth mindset moderated the relation between the time elapsed since their latest NRP course and their performance on the neonatal resuscitation tasks presented in the RETAIN game. Figure **4** displays the adjusted effect of the Last NRP Course on the Number of Tries (y axis) across all continuous values of Growth Mindset (x axis). The region of significance is determined by the locations where the upper and lower bounds of the 95% confidence interval intersect zero (i.e., for any values of the moderator for which the confidence bands do not contain zero, the effect of the predictor on the outcome is significantly different from zero). The confidence band crosses zero at m^*^ = 0.03 in Figure **4**, with a vertical dotted line marking the boundary between regions of significance and non-significance. Thus, the region to the left of the vertical dotted line represents the region of significance.

**Figure 3:**
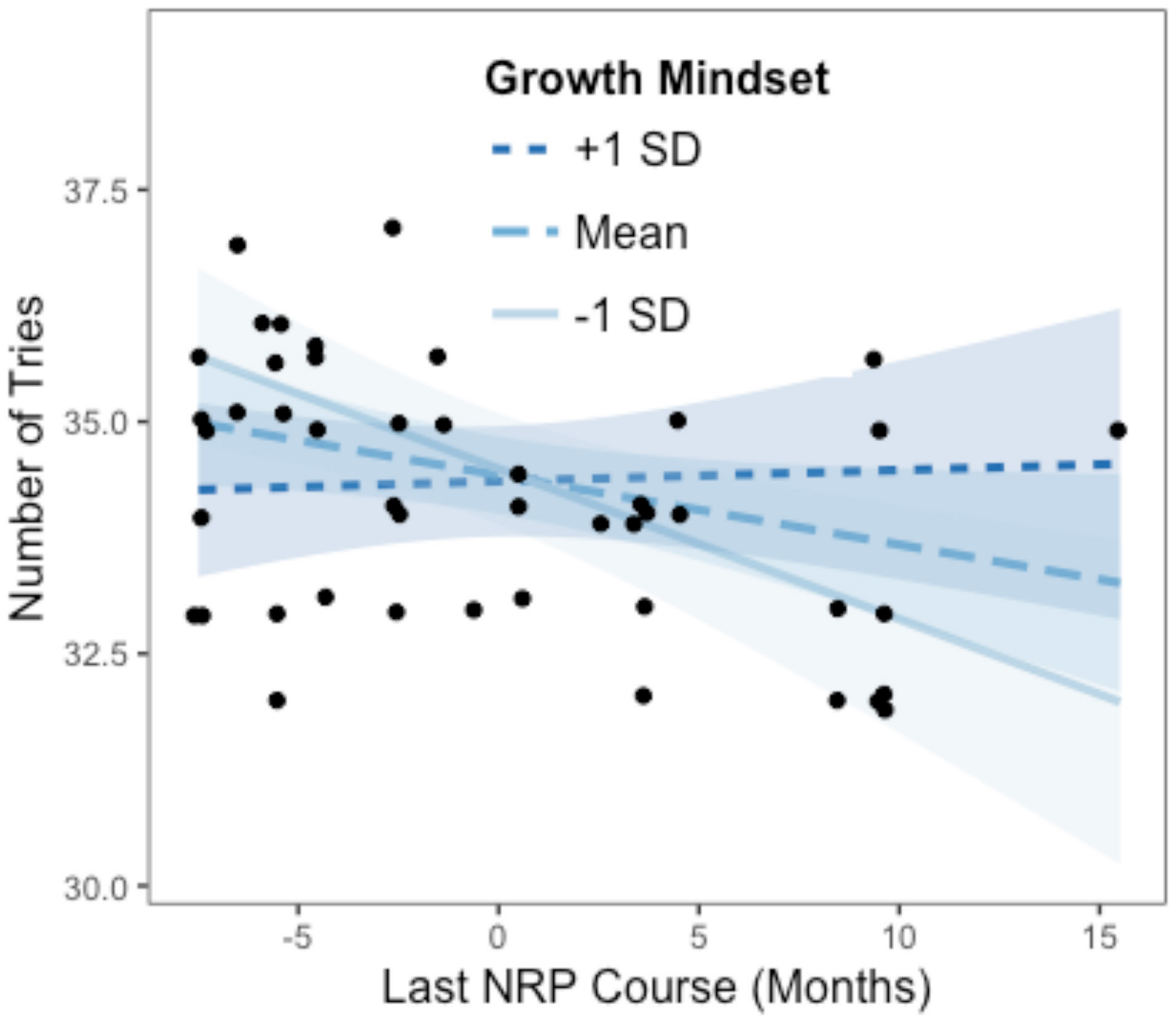
Johnson-Neyman interaction effect of the linear regression model. The x-axis represents the centered variable Last NRP Course and the y-axis represents the number of tries in the RETAIN game. Participants who took an NRP course more recently (i.e., when the value of Last NRP Course on the x axis is lower than zero which represents the average of 8.69 months) required significantly more tries to complete the game than the rest of the participants, but only when they endorsed lower levels of Growth Mindset (i.e., at the mean or one standard deviation below the mean).

**Figure 4:**
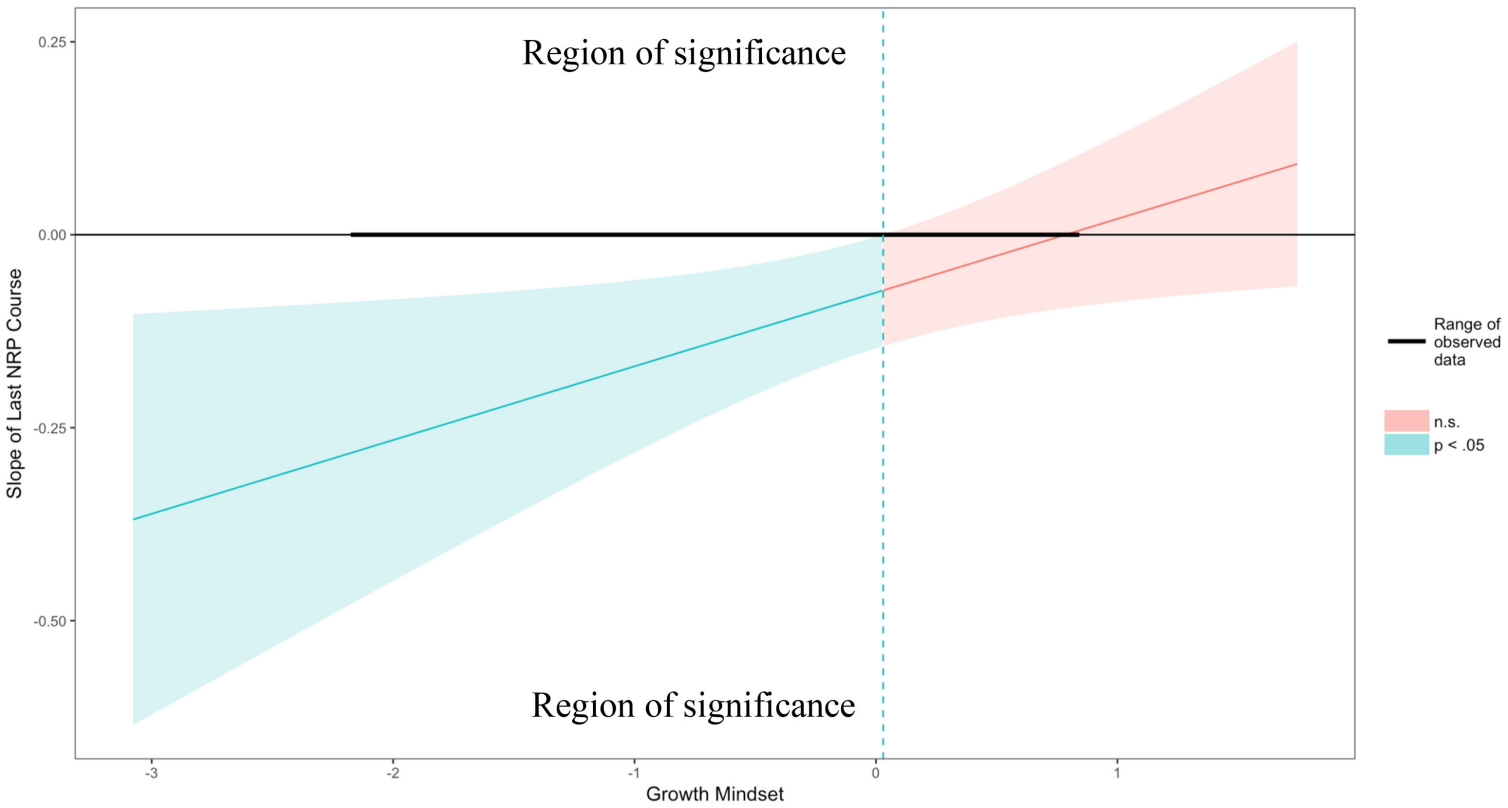
Probing interactions with the Johnson-Neyman (J-N) technique for continuous variables. The y axis represents the conditional slope of Last NRP Course predictor, while the x axis represents units of standard deviation of the Growth Mindset moderator. The plot shows where the conditional slope differs significantly from zero. The light blue area on the left of the vertical dotted line between the 95% confidence bands represents the region of significance where the effect of the Last NRP Course on the Number of Tries is significantly moderated by Growth Mindset (i.e., this effect only exists when Growth Mindset is lower than or equal to 0.03 represented by the vertical dotted line). The dark horizontal line indicates the actual range of Last NRP Course observed in the sample data: [−2.17, 0.83].

Results showed that there was a significant interaction of Last NRP Course and Growth Mindset in predicting Number of Tries (*b* = 0.09, SE = 0.04, beta = 0.32, *t* = 2.25, *p* = 0.03). This means that every time a person's Growth Mindset score increases by 1, the adjusted effect of Last NRP Course on Number of Tries (i.e., the y axis in Figure 4) increases by 0.09. Thus, participants who took an NRP course up to ~8 or 9 months before the current study (i.e., the average Last NRP course value) completed the game in significantly more tries than the rest of the participants, but only when they endorsed lower levels of growth mindset, as shown in Figure 3; they also made fewer mistakes in the game as their growth mindset increased.

The Figure 3 plot shows the individual data points to better understand how the fitted lines relate to the observed data and it provides a sense of the precision of the estimates by plotting 95% confidence bands based on robust standard error calculations. Results of the analysis reveal that the slope of Last NRP Course (y axis) is statistically significantly different from zero at *p* < 0.05 when Growth Mindset (x axis) is outside the [0.03, 5.91] interval (i.e., all values of the Growth Mindset moderator outside of this interval will have a significant slope for the Last NRP Course predictor). Also, the effect of Last NRP Course on the Number of Tries only exists when Growth Mindset is lower than 0.03, in which case the conditional slope of the Last NRP Course is statistically significantly different than zero, as shown in Figure 4. For example, when growth mindset is zero (i.e., the mean of the sample, as the variable was centered), the estimated slope of Last NRP Course is −0.075, S.E. = 0.04, *p* = 0.04. When the growth mindset is −0.91 (i.e., one standard deviation below the sample mean), the estimated slope of Last NRP Course is −0.162, S.E. = 0.05, *p* = 0.004. Finally, the slope is not significant when growth mindset is 0.91 (i.e., one standard deviation above the sample mean), with the estimated slope of Last NRP Course being 0.01, S.E. = 0.05, *p* = 0.81. The Figure 4 plot shows where the conditional slope differs significantly from zero. From the point Growth Mindset (the moderator) is lower than 0.03, the slope of Last NRP Course (the predictor) is statistically significantly different from zero. The upper bound for this interval (5.91) is so far outside the observed data that it is not plotted. Thus, the Last NRP Course has no effect on the outcome variable except when Growth Mindset is lower than 0.03 (we do not interpret the upper boundary of the interval because the dataset does not contain any values near it). For lower values of Growth Mindset (i.e., 0.03 or approximately lower than the mean Growth Mindset), the higher the growth mindset, the more strongly Last NRP Course negatively predicts game performance. Thus, results suggest that participants who completed the NRP course within 8–9 months before this study made statistically significantly more mistakes in the game compared to their counterparts who completed the course between 9 and 24 months prior to the study, but only when they endorsed a lower rather than a higher growth mindset, as also shown in Figure 4.

**Figure 5:**
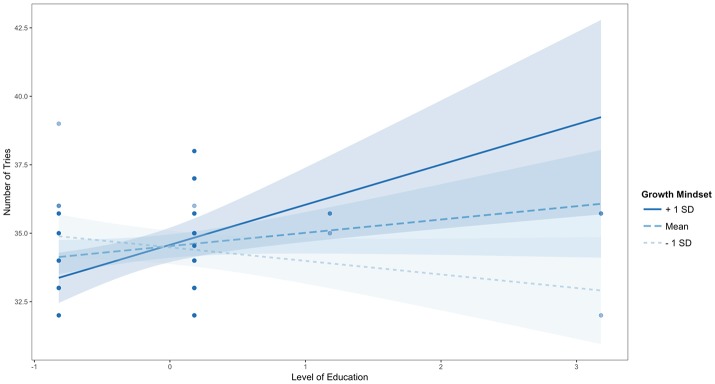
Johnson-Neyman interaction effect of the linear regression model. The x-axis represents the centered variable Level of Education and the y-axis represents the number of tries in the RETAIN game. Participants with lower levels of education completed the game in fewer tries than participants with higher levels of education but only when they endorsed higher levels of growth mindset (i.e., one standard deviation above the mean).

**Figure 6:**
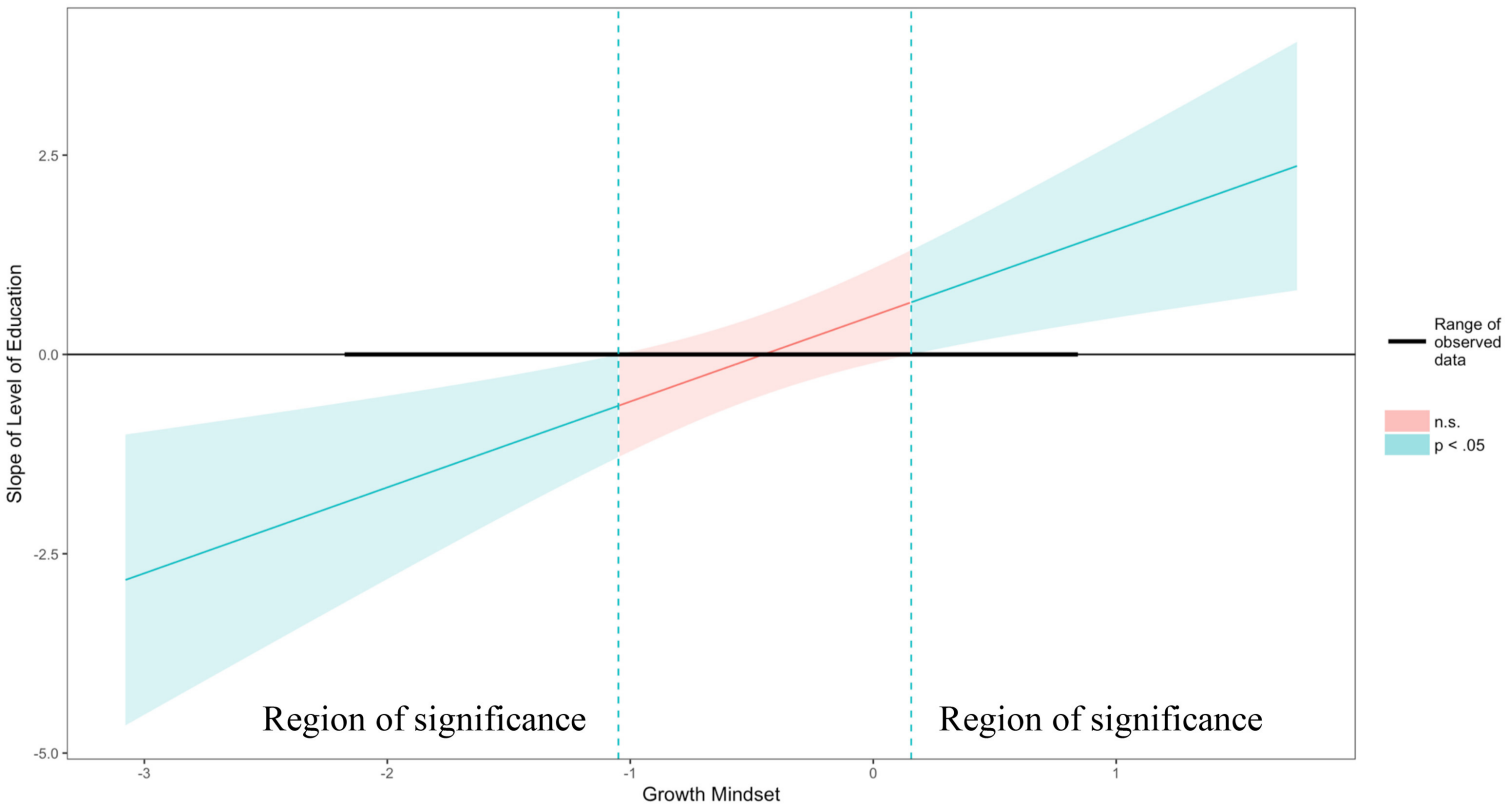
Probing interactions with the Johnson-Neyman (J-N) technique for the Level of Education predictor and the Growth Mindset moderator. The plot shows two light blue regions of significance where the effect of the Level of Education on the Number of Tries is significantly moderated by Growth Mindset (i.e., this effect only exists to the left of the first vertical dotted line and to the right of the second vertical dotted line). The dark horizontal line indicates the actual range of Level of Education observed in the sample data.

The model using Level of Education and Growth Mindset to predict Number of Tries was also significant [*F*_(3, 46)_ = 3.62, Adjusted R-squared = 0.14, *p* < 0.05] with an interaction (*b* = 1.08, SE = 0.33, beta = 0.52, *t* = 3.24, *p* < 0.01) but no main effect for Level of Education (*b* = 0.48, SE = 0.29, beta = 0.26, *t* = 1.65, *p* = 0.11) and no main effect for Growth Mindset (*b* = 0.05, SE = 0.24, beta = 0.03, *t* = 0.20, *p* = 0.84). The Johnson-Neymar technique identified two regions of significance illustrated as the light blue areas in Figure 6: the slope of Level of Education (y axis) is statistically significantly different from zero at *p* < 0.05 when Growth Mindset (x axis) is outside the [−1.05, 0.16] interval. This means that, when Growth Mindset is lower than −1.05 standard deviations from its mean or higher than 0.16 standard deviations from its mean, every time a person's Growth Mindset score increases by 1, the adjusted positive effect of Level of Education on Number of Tries (i.e., the y axis in Figure 5) increases by 1.08. Thus, participants with a diploma or a Bachelor's degree made significantly more mistakes in the game compared to their counterparts with more advanced degrees, but only when participants endorsed lower levels of growth mindset (i.e., lower than the growth mindset average of this sample); also, as their level of growth mindset increased, their mistakes also decreased, as shown in Figures 5, 6. For example, when growth mindset is zero, the estimated slope of Level of Education (0.49, SE = 0.30, *p* = 0.11) is not significant. Also, when the Growth Mindset is −0.91 (i.e., one standard deviation below the sample mean), the estimated slope of Level of Education (−0.50, SE = 0.29, *p* = 0.10) is not significant. Finally, the slope is significant when Growth Mindset is 0.91 (i.e., one standard deviation above the sample mean), with the estimated slope of Level of Education being 1.47, SE = 0.52, *p* < 0.05.

Similar analyses using Years of Experience instead of Last NRP Course yielded a non-significant model [*F*_(3, 46)_ = 0.21, Adjusted R-squared = −0.05, *p* = 0.89] with no interaction (*b* = 0.01, SE = 0.03, *t* = 0.42, *p* = 0.67), no main effect for Years of Experience (*b* = 0.01, SE = 0.03, *t* = 0.37, *p* = 0.71), and no main effect for Growth Mindset (*b* = −0.15, SE = 0.26, *t* = −0.59, *p* = 0.56).

## Discussion

Current neonatal resuscitation training is performed on a mannequin in a specialized facility (e.g., Simulation Lab) where participants are trained by instructors specialized in SBME. Consequently, SBME in its current form is time and cost intensive and, therefore, not offered routinely, regularly, or in all health-care facilities (16). The current physical SBME training is initially effective but frequent refresher training sessions are necessary for a trainee to retain the required knowledge (14, 15, 52). With the current physical training methodologies, frequent refresher sessions are cost-prohibitive. Accordingly, the current certification requirements demand only a single 4–h SBME refresher course every 2 years to remain certified in neonatal resuscitation. While SBME has been shown to improve performance initially after training (14, 15), both cognitive and technical skills significantly deteriorate within months (16).

The results of the current study suggest that Growth Mindset is a moderator of the relation between Last NRP Course and performance in a computer game training simulator, which could help explain temporal differences in the effectiveness of NRP training that were not detected by correlation analyses alone. Participants who took the NRP course within the past 8 or 9 months made significantly more mistakes in a training simulation game than the rest of the participants, but only when they endorsed lower levels of growth mindset. This result echoes other studies showing a significant deterioration of resuscitation knowledge and skills at 6–8 months after the completion of the NRP course (7). Growth Mindset also moderated the relation between Level of Education and Number of Tries, showing that individuals with lower level of education make more mistakes in the game in comparison with their peers with more advanced levels of education but only when they endorse lower levels of growth mindset. Taken together, these results highlight the importance of factors such as growth mindset in moderating the relation between performance and education.

It is possible that the observed differences in performance depend on individual differences and variables not currently measured, such as age, stress, mood, anxiety, test fatigue, motivation at work, or job performance. This limitation will be addressed in part in a follow-up study that aims to collect age information and a range of biometric data from the participants. It is also possible that participants did not take too seriously the low-stakes environment of a training simulation computer game and they did not exhibit maximal (i.e., test) behaviors. Finally, other variables we measured, such as clinical role (e.g., nurse, respiratory therapists, etc.) and the number of years of clinical experience in neonatal resuscitation, did not yield any significant influences on performance or on the relation between NRP timeline and performance.

These results are in accord with the NRP program caution that completion of the course does not equate proficiency in infant resuscitation in a clinical environment. Specifically, the NRP course is necessary, but not sufficient in achieving high performance. Perhaps participants who completed the course up to 8 or 9 months before the study need more time and deliberate practice opportunities to consolidate their knowledge through intensive practice sessions. As the bridging between program completion and clinical competence is still a matter of research and evaluation, simulation computer games constitute a promissory avenue for consolidating the skills acquired in NRP training. In the future, we also plan to augment the RETAIN game with more collaborative features that would simulate more intentionally the diversity of skills and richness of communication present in neonatal resuscitation teams.

## Conclusions and implications

The current study examines empirically the relation between health-care providers' task performance and the time elapsed since their latest NRP course and found that growth mindset moderates this relation. Specifically, the participants who took the NRP course within the past 8 or 9 months made more mistakes in a training simulation computer game than the rest of the participants but only when they endorsed lower levels of growth mindset. To our knowledge, this is a first demonstration that mindset moderates the relation between NRP training and performance. This study constitutes a first step in clarifying the relation between NRP courses and performance, and more research is needed to examine other possible factors that could moderate this relation. Recently, brief social-psychological interventions have been gaining increased attention as approaches for increasing motivation for persistence (53), performance (51, 54), and completion (55). Thus, some implications of this research include growth mindset interventions that aim to shift the way individuals attribute professional success from fixed (e.g., innate abilities) to more malleable factors (e.g., effort). These interventions may engender a mindset that views intelligence as malleable and open to growth, as opposed to immutable (53, 56). Future studies may combine growth mindset interventions with increased opportunities to practice skills in simulation sessions to help health-care providers achieve better performance, especially shortly after taking a refresher NRP course.

## Author contributions

GS, MC, and MB: conception and design; CF, GS, MB, and MC: collection and assembly of data; MC, GS: analysis and interpretation of the data; MC, GS, MB, and CF: drafting of the article; MC, GS, MB, and CF: critical revision of the article for important intellectual content; GS, MC, MB, and CF: final approval of the article.

### Conflict of interest statement

The authors declare that the research was conducted in the absence of any commercial or financial relationships that could be construed as a potential conflict of interest.

## Abbreviations

NRP: Neonatal resuscitation program
SBME: Simulation-based medical education
HCP: Health-care providers.
